# Sleep-Disordered Breathing and Hypertension—A Systematic Review

**DOI:** 10.3390/jcm14093115

**Published:** 2025-04-30

**Authors:** Ayman Battisha, Amrit Kahlon, Dinesh K. Kalra

**Affiliations:** Division of Cardiology, Department of Medicine, University of Louisville, 201 Abraham Flexner Way, Suite 600, Louisville, KY 40202, USA; a0batt07@louisville.edu (A.B.); a0kahl02@louisville.edu (A.K.)

**Keywords:** sleep-disordered breathing, hypertension, cardiovascular disease, resistant hypertension, obstructive sleep apnea, cardiovascular prevention

## Abstract

**Background/Objectives:** Sleep-disordered breathing (SDB), historically referred to as “sleep apnea syndrome”, particularly obstructive sleep apnea (OSA), is an independent risk factor for hypertension (HTN), stroke, heart failure, arrhythmias, and other cardiovascular disorders. Despite the well-established link between OSA and HTN and its high occurrence in cardiovascular disorders, the focus on the complex OSA–HTN axis is often overlooked or inadequately managed, which might explain the lack of notable improvements in cardiovascular outcomes for this patient population. Understanding the complex relationship between OSA and HTN is crucial due to its significant implications for clinical practice and public health. **Methods:** Using an expanded list of relevant MeSH terms, including “sleep-disordered breathing” and “sleep apnea syndrome”, and following the PRISMA model, peer-reviewed articles were systematically selected. Studies published from January 2000 through December 2024 were identified and screened based on predefined inclusion and exclusion criteria. **Results:** This review emphasizes both OSA’s independent and interaction effects on cardiovascular health and outcomes across different populations. It identifies key factors mediating the association between OSA and HTN. **Conclusions:** Multimodal management, including continuous positive airway pressure and lifestyle modification, is essential for treating hypertension related to OSA. Effective management of the OSA–HTN relationship is vital to improving cardiovascular outcomes.

## 1. Introduction

Sleep is generally a time of cardiovascular relaxation, especially during the nonrapid eye movement (NREM) sleep stage when the cardiovascular system has reduced arterial blood pressure, heart rate, metabolic rate, and cardiac workload. Any disruption from standard sleep patterns, such as poor quality and quantity of sleep, may lead to a negative cardiovascular cascade that increases the incidence and burden of cardiovascular disease (CVD) [1]. 

Sleep-disordered breathing (SDB) is a spectrum of abnormal respiration patterns in patients during sleep, resulting in intermittent hypoxia and sleep fragmentation. Obstructive sleep apnea (OSA) is the most prevalent form among these conditions, as it affects approximately a third of the general population and more than 40% of the patients suffering from CVD [2,3]. The recurrent episodes of upper airway collapse are due to reduced muscle tone during sleep. These intermittent blockages can be complete (apneas) or partial (hypopneas), leading to a cycle of intermittent low oxygen levels in the blood, disturbances in autonomic nerve function, and disrupted sleep patterns. OSA has developed into a primary public health concern globally because of its prevalence and significant effects on the health domain. Current evidence suggests that cyclical oxygen desaturation and reflexive sympathetic hyperactivity linked to OSA are associated with the mechanisms leading to the onset of hypertension and various downstream cardiovascular diseases [2,3].

Hypertension (HTN) remains one of modern society’s leading global health concerns, accounting for 10.8 million deaths, translating to 19.2% of all deaths in 2019 and 9.3% of disability-adjusted life-years lost worldwide [4]. HTN is a significant risk factor for cardiovascular morbidity and mortality. Although antihypertensive medications are readily available in contemporary health facilities, many individuals globally remain inadequately controlled, with a substantial proportion of cases being resistant to standard treatments [5]. The emergence of OSA-related hypertension, which is mainly nocturnal with a non-dipping pattern, has created a unique medical challenge that needs a more profound and comprehensive understanding of this co-existence to enhance clinical management.

OSA and HTN have a complex relationship comprising risk factors, mechanisms, and clinical manifestations. Previously published studies suggest that OSA patients are at a high risk of developing HTN, irrespective of other conditions, such as age, obesity, and gender [6,7,8,9]. Several pathophysiological mechanisms and shared relationships exist between OSA and HTN, including intermittent hypoxia, sympathetic nervous system activation, inflammation, oxidative stress, endothelial dysfunction, and alterations in intrathoracic pressure. This systematic review elaborates on the latest scientific developments linking SDB to HTN, focusing on shared risk factors, underlying pathophysiological pathways, clinical features, and treatment modalities.

## 2. Epidemiology

OSA and HTN frequently coexist, demonstrating a bidirectional relationship and a dose-response correlation [10,11]. This relationship is particularly evident in treatment-resistant hypertension (TRH) and masked hypertension [11,12]. Hypertension is highly prevalent, partly because it often presents no symptoms until hypertension-mediated multi-organ damage (HMMOD) becomes apparent. It remains a significant cause of preventable morbidity and mortality worldwide, accounting for approximately 10 million deaths in 2015 [13].

Multiple cross-sectional studies confirm that an increased prevalence of OSA is an important risk factor for HTN, independent of age, gender, and obesity [3]. In 2023, the World Health Organization (WHO) estimated that approximately 1.3 billion adults aged 30–79 years worldwide have hypertension, with about half unaware of their condition. The estimated prevalence of OSA in North America is 20–30% in males and 10–15% in females [14]. The gender disparity narrows post-menopause, possibly due to hormonal changes. Certain racial/ethnic groups, particularly African Americans and Hispanics, have higher prevalence rates of OSA and HTN compared to Caucasians, even when accounting for confounders like obesity or income [14].

There is also a disparity in the epidemiological distribution of HTN based on age, sex, race, and region [15]. For example, HTN affects African Americans more than any other race, with higher cases originating at earlier ages and with greater severity. Additionally, data have shown that the incidence of HTN rises with age, with more than 50 percent of the elderly population having the disorder and nearly two-thirds of people over the age of 60 [15]. The profile of these demographic factors, coupled with the epidemiology of SDB, points to the importance of designing specific screening and targeted interventions in this patient population.

## 3. Screening and Definitions

SDB is a cluster of diseases characterized by breathing disorders during sleep, causing intermittent hypoxia, sleep fragmentation, and a variety of comorbidities. It is important to note that in some patients, the classic OSA-related risk factors, such as obesity and increased neck circumference, might be absent. Sleep apnea may not be caused by upper airway collapse but by other non-anatomic mechanisms, such as reduced responsiveness of the upper airway, ventilatory control instability, and decreased sleep arousal threshold [16]. Regardless of the mechanism, partial or complete recurrent upper airway obstruction during sleep results in apneas or hypopneas [17]. These episodes result in oscillations between hypoxia and hyperpnea and the repetitiveness of sleep awakenings, which cause excessive daytime sleepiness and cardiovascular complications such as HTN. Other types of SDB include central sleep apnea (CSA), associated with cessation of respiratory effort due to the failure of the brain’s respiratory control centers, and complex sleep apnea syndrome (CompSAS), which is OSA associated with CSA. Hypoventilation Syndrome is another form of SDB related to inadequate sleep ventilation, possibly due to obesity or neuromuscular diseases [18]. Even though OSA is the most common subtype and is currently under the most intense investigation, the entire spectrum of SDB also has many risks related to the development of cardiovascular diseases.

The current guidelines for hypertension screening suggest a frequency of once every 5 years for individuals with a blood pressure reading of less than 120/80 in a clinical setting. Screenings should occur every 3 years for those with readings between 120–129/80–84, and those with blood pressure between 130–139/85–89 should undergo annual screening [10,11]. HTN is diagnosed based on blood pressure readings, with the American College of Cardiology/American Heart Association (ACC/AHA) classifying it as a systolic blood pressure (SBP) of 130 mm Hg or higher, or a diastolic blood pressure (DBP) of 80 mm Hg or higher [19]. Blood pressure (BP) is typically measured using a calibrated sphygmomanometer. Ambulatory blood pressure monitoring (ABPM) is essential in diagnosing hypertension, especially when suspecting white-coat HTN, masked HTN, and nocturnal HTN, particularly in patients with suspected SDB (Table 1).

ABPM detects BP variations during the day and at night. Therefore, it will also detect the nocturnal rises in blood pressure typical of patients with OSA [20]. The non-dipping pattern of blood pressure, in which blood pressure does not decrease at night, is an added risk to the cardiovascular system and is unique to patients with OSA. ABPM monitoring is also crucial for managing pediatric SDB and HTN, as it guides treatment decisions and prognosis based on severity. The severity of HTN has significant clinical implications, as higher blood pressure levels are directly associated with an increased risk of cardiovascular and renal complications. Consequently, treatment strategies should correlate with the severity of HTN, with more aggressive interventions recommended for those with higher blood pressure readings or evidence of target organ damage.

Screening for OSA is indicated in any patient with daytime sleepiness, difficult-to-treat hypertension, and metabolic syndrome. In-patient polysomnography (PSG) is the current gold standard for diagnosing OSA and its severity [3]. It includes monitoring multiple variables simultaneously by recording airflow, respiratory efforts, oxygen saturation, electroencephalogram (EEG), electrocardiogram (ECG), and muscle activity during sleep [18]. Unattended home sleep apnea testing (HSAT) with type III monitors provides a pragmatic, lower-cost alternative to in-lab PSG for adults who have a high pre-test probability of moderate-to-severe OSA. Randomized trials demonstrate that HSAT-based pathways yield symptom relief and blood-pressure reductions comparable to PSG when positive HSAT results are coupled with auto-titrating CPAP therapy [21,22]. Nevertheless, because HSAT omits electroencephalography, it underestimates the apnea–hypopnea index (AHI) and misses arousal-only events; pooled data on single-channel, type IV devices show a sensitivity of ≈76 percent and specificity of ≈44 percent, making them unreliable for excluding mild disease [23]. The American Academy of Sleep Medicine, therefore, recommends PSG when HSAT is negative or inconclusive despite strong clinical suspicion, or when significant cardiopulmonary, neuromuscular, or central-apnea comorbidities are present [23,24]. Building on the pragmatism of HSAT, consumer wearables now offer an even less intrusive screening option. In a PSG-controlled cohort, Galaxy Watch 4 and Apple Watch 7 identified OSA (AHI ≥ 5) with 82.9/75.8 percent and 71.1/62.5 percent sensitivity/specificity, respectively, yielding AUC values ≈0.80 [25]. Validation of an under-mattress pneumatic sensor against PSG demonstrated 86 percent sensitivity and 88 percent specificity for moderate disease [26]. A recent systematic review confirmed substantial inter-device variability and concluded that no wearable yet matches PSG for routine diagnosis, although Samsung’s Galaxy Watch sleep-apnea algorithm received FDA de novo authorization in 2024 as an over-the-counter screening tool [27]. Therefore, we best assess OSA diagnosis and severity using PSG parameters, such as sleep architecture, respiratory events, and their effects on oxygenation and cardiac function. We grade the severity of OSA based on the AHI, where apnea refers to a complete cessation of airflow for more than 10 s, and hypopnea refers to a reduction in airflow to less than 50% of normal levels. An AHI index of 5–15 indicates mild OSA severity; an index of 16–30 indicates moderate severity; and an index greater than 30 indicates severe OSA [3,14]. The Oxygen Desaturation Index (ODI), the number of times a person’s oxygen level drops during sleep, is another measure of disease severity (Table 2) [14].

Other studies have argued that we should assess OSA severity using measures beyond AHI. They proposed alternatives like the Ventilatory Burden (VB), which measures the proportion of small breaths overnight, and the Baveno classification, a multicomponent grading system [28]. These methods aim to capture OSA phenotypes and airflow reductions independent of hypoxemia or arousal, providing a more comprehensive characterization of OSA severity and addressing some of the limitations of the traditional AHI metric. Accurate categorization of OSA severity and phenotypes has broader clinical significance since there appears to be a dose-response correlation between the severity of OSA and the onset of hypertension [3,29]. Results from other studies suggest that higher AHI and specific OSA phenotypes, such as younger age, excessive daytime sleepiness, uncontrolled BP, and severe OSA-related oxygen desaturations, may predict benefits from continuous positive airway pressure (CPAP) therapy in terms of BP management [29].

## 4. Materials and Methods

### 4.1. Research Design

This research used a systematic literature review to analyze the link between sleep-disordered breathing (SDB) and hypertension. A systematic review is the most suitable approach for synthesizing research findings to understand patterns and trends across multiple interrelated studies. To enhance the methodological quality of the review, we used the PRISMA checklist for the inclusion of articles in systematic reviews and meta-analyses.

### 4.2. Search Strategy

The literature review process was performed using several electronic databases, including PubMed, Scopus, and Web of Science. These databases were particularly chosen since they provided a wide coverage of medical, as well as scientific, research. The search strategy incorporated an expanded set of keywords and MeSH terms, including “sleep-disordered breathing”, “sleep apnea syndrome”, “obstructive sleep apnea”, “central sleep apnea”, “hypertension”, and “high blood pressure”. Boolean operators like AND and OR were used to refine the search process and ensure comprehensive coverage of relevant literature. The search was conducted by two independent reviewers to enhance rigor and reduce bias. Additionally, the search period was explicitly defined as January 2000 through December 2024, ensuring clarity and reproducibility of the search methodology. Only peer-reviewed articles written in English and published within this timeframe were included to ensure the incorporation of recent findings.

### 4.3. Selection Criteria

To ensure the relevance and quality of the studies included in the review, specific inclusion and exclusion criteria were established:

#### 4.3.1. Inclusion Criteria

- Studies that explicitly examined the relationship between sleep-disordered breathing (including sleep apnea syndrome and obstructive sleep apnea) and hypertension.
- Peer-reviewed empirical studies, including randomized controlled trials, cohort studies, and cross-sectional studies.
- Articles published in English between January 2000 and November 2024.

#### 4.3.2. Exclusion Criteria

- Non-empirical studies, such as reviews, commentaries, or editorials.
- Articles not available in full text.

### 4.4. Search Process

**Figure 1:**
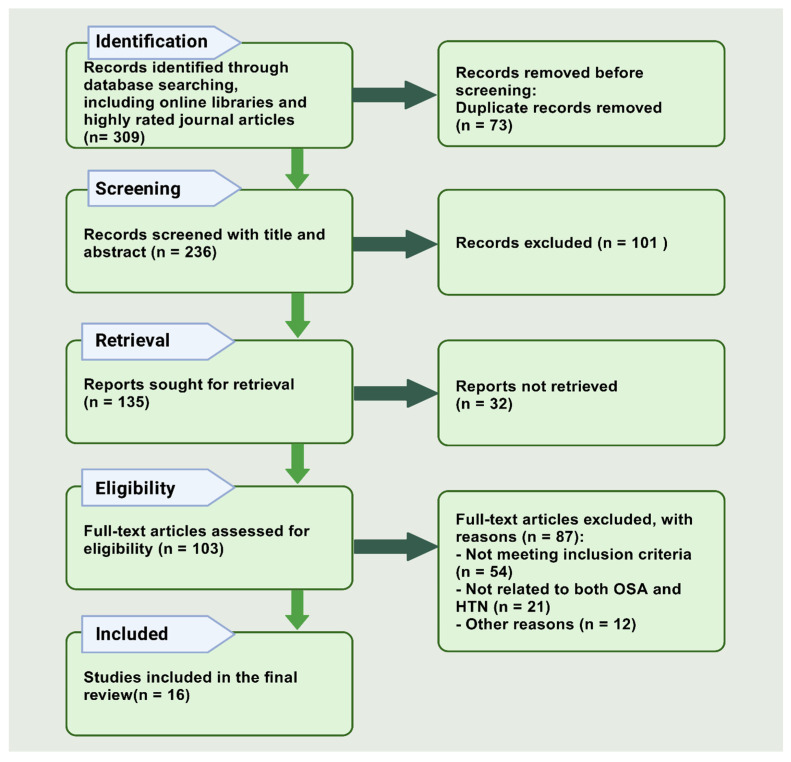
PRISMA chart flow diagram for updated systematic reviews, which included searches of databases.

We began the systematic literature review by defining the area of interest and conducting a database search, identifying 309 records from reliable online libraries and highly rated journals. After removing 73 duplicate records, we had 236 remaining for screening by title and abstract. We excluded 101 records, leaving 135 reports for further review. However, only 32 reports were unmatched and not retrieved, resulting in 103 full-text articles for eligibility assessment. During this stage, we excluded 87 articles for various reasons: 54 did not meet the inclusion criteria, including 21 that were not relevant to SDB and HTN, and for other reasons, 12 studies were excluded, such as insufficient data, incomplete recording, missing key outcome measures, presenting incomplete results, or lack of detailed methodology. We also excluded articles with poor study design, high risk of bias, or lack of peer review. Ultimately, we selected 16 articles for review (Figure 1).

### 4.5. Extraction and Management of Data

Electronic and paper copies of the 16 selected studies were reviewed, and data were systematically extracted using a supportive data-extraction form. Information extracted from the selected studies consisted of article characteristics: authors, year of publication, the kind of research, number of participants, age, gender, and health state of the participants, measures of SDB and hypertension, and key findings. All the extracted data were entered into an Excel sheet for organization and analysis. (Table 3).

### 4.6. Results

The reviewed studies encompass various research designs, including observational, prospective cohort, cross-sectional, experimental interventional, and randomized controlled trials. Participant numbers range from as few as 10 (e.g., Adam Witkowski et al., 2011 [34]) to as many as 11,623 (e.g., Xiaoyu Li et al., 2020 [46]), reflecting differences in researchers’ interests and chosen methodologies. Age groups include children, pregnant women, older adults, and patients with various conditions such as pulmonary arterial hypertension or resistant hypertension. Researchers have employed several metrics for SDB and hypertension, including AHI, ODI, and 24 h ambulatory blood pressure monitoring. These studies collectively address gender differences in the effects of SDB on hypertension across diverse age groups and associated comorbidities, demonstrating both direct and moderating impacts on cardiovascular and overall health.

## 5. Discussion

### 5.1. Shared Risk Factors for Both HTN and SDB

Aging is a significant risk factor for both HTN and OSA. Other contributing mechanisms include impaired arterial wall elasticity, increased vascular resistance, and alterations in the balance of the autonomic nervous system [38]. As with other respiratory system components, the upper airway structures undergo anatomical changes with age; the neural drive to the upper airway muscles diminishes, and fat is deposited around the neck and in the pharynx, making older adults more susceptible to OSA. In patients with OSA, increased age is a significant risk factor for HTN, OSA, and the cumulative incidence of cardiovascular disease [47,48].

There is substantial evidence linking obesity, particularly central obesity, with both HTN and OSA. Excess body fat, especially in the abdominal area, leads to insulin resistance, dyslipidemia, and inflammation, contributing to HTN [6]. In the case of OSA, obesity causes fat deposition in the upper airway, increasing the likelihood of airway closure during sleep [49]. Additionally, obesity is a risk factor for metabolic syndrome, a cluster of disorders that include HTN, dyslipidemia, insulin resistance, and central obesity. By exacerbating the severity of OSA and increasing the risk of cardiovascular diseases, metabolic syndrome contributes to the worsening of outcomes associated with both conditions. It is now well-established that weight-loss interventions improve blood pressure control and OSA severity, underscoring the role of obesity management in treating these diseases. In a study that examined the association between probable OSA (pOSA) and HTN by race, when the sample models were stratified by both race and weight, compared to overweight whites, pOSA predicted a four-fold and two-fold increased risk of HTN among overweight Black/African Americans and obese Hispanic/Latino participants, respectively. The results of this study highlight the importance of targeted OSA screening in these patient populations [50].

Sex significantly influences the occurrence, manifestation, and prognosis of both HTN and OSA. OSA is more prevalent among men than women, with a prevalence ratio of 2:1 to 3:1 in the general population [51]. Studies suggest that the gender disparity might be due to anatomical differences in the upper airway, fat distribution, and hormonal factors. Although some studies found no differences in the incidence of OSA in women, there is an increase in the incidence of the disease post-menopause, likely due to hormonal influences that impact airway integrity. On the other hand, HTN is more common in men during early adulthood but affects women more frequently in later life stages, especially post-menopause [52]. Additionally, the presentation of these disorders differs between genders; female patients with OSA are more likely to present with symptoms such as insomnia and fatigue, which are uncharacteristic of the usual disordered breathing during sleep [53]. Understanding these sex-specific differences is crucial for effective screening, diagnosis, and management of both HTN and OSA.

The prevalence and manifestation of both HTN and OSA also depend on race and ethnicity. For example, African Americans have an increased prevalence of HTN and are more likely to present with early-onset, complicated, or treatment-resistant phenotypes compared to Caucasians [15]. Genetic differences, including variations in sodium processing and environmental and socioeconomic factors, partly contribute to this disparity. Similarly, there is an increased incidence of OSA in African American and Hispanic communities, considering obesity and other variables [54]. Differences in the head and neck structure and adipose tissue distribution may also play a role. Genetic predispositions also play a role in the risk of HTN and SDB, as several polymorphisms are known to be associated with these conditions. The issues of race, ethnicity, and genetics suggest that more targeted approaches are required to treat or prevent these conditions in different communities.

Genetics plays a crucial role in HTN and OSA, with substantial evidence in the literature supporting the hereditary patterns of these diseases. Family-based research has shown that individuals with a family history of HTN or OSA are more likely to develop these conditions than those without such a history, indicating a hereditary component [55]. Several studies have linked numerous genetic polymorphisms to HTN, including genes encoding for the renin–angiotensin–aldosterone system (RAAS), sodium-channel transporters, and endothelial function. Similarly, there are genetic predictors of OSA, such as those related to craniofacial morphology, upper-airway muscle contraction, and inflammation [17]. The genetic connection between HTN and OSA may explain the interaction between these two conditions in different individuals. Understanding the molecular mechanisms that govern the development of HTN and OSA is critical for risk assessment and the development of targeted precision medicine in patients with OSA and HTN.

**Figure 2:**
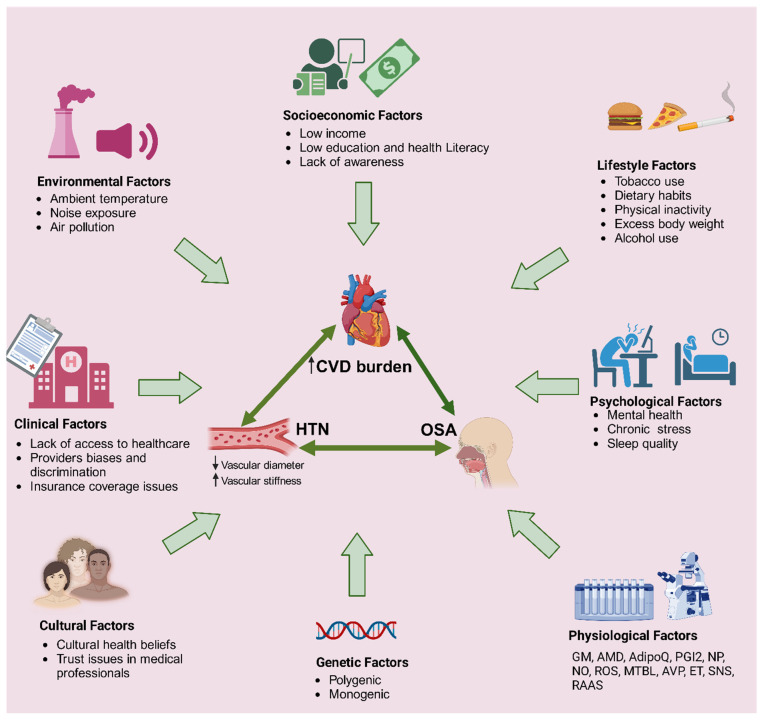
Mechanisms involved in the incidence of CVD and the shared pathophysiological risk factors of OSA and HTN. Several biological and environmental factors contribute to the incidence of HTN and CVD. Abbreviations: RAAS = renin-angiotensin-aldosterone system, ET = endothelin, AVP = arginine vasopressin; MTBL = metabolome, ROS = reactive oxygen species, NO = nitric oxide, NP = natriuretic peptides, PGI_2_ = prostacyclin, AdipoQ = Adiponectin, ADM = adrenomedullin, GM = Gut microbiota, HTN = hypertension, OSA = obstructive sleep apnea, CVD = cardiovascular disease; SNS = sympathetic nervous system; ↑ = increase; ↓ = decrease.

In addition to non-modifiable risk factors, lack of physical activity, high-calorie diets, alcohol consumption, and smoking have historically been identified as risk factors for HTN and OSA (Figure 2). Lack of physical activity is a primary cause of obesity and metabolic dysfunction underlying both conditions. Studies consistently show that high sodium intake, high intake of saturated fats, and processed foods contribute to HTN [56]. Conversely, foods and nutrients with proven protective effects include fruits, vegetables, and whole grains. Alcohol intake is a risk factor for both HTN and OSA because it worsens airway conditions during sleep and increases blood pressure. Smoking also contributes to inflammation, oxidative stress, and endothelial dysfunction, which contribute to the development of HTN and OSA [57,58]. Lifestyle modifications aimed at reducing these risk factors, such as increased physical activity, improved diet, reduced alcohol consumption, and smoking cessation, are crucial for preventing and treating both conditions and reducing CVD burden.

### 5.2. Pathophysiology Linking SDB and HTN

Intermittent hypoxia (IH), a key feature of OSA, results from cyclic collapses of the upper airway and subsequent temporary hypoxemia during sleep. This cycle of hypoxia and reoxygenation triggers various pathophysiological changes, leading to the development of HTN [59]. Hypoxia-induced stress causes the formation of reactive oxygen species (ROS), which, in turn, activate numerous inflammatory mediators during intermittent hypoxia. Intermittent hypoxia in sleep apnea also increases oxidative stress, generating inflammation and causing endothelial dysfunction, increased vascular stiffness, and enhanced vasoconstriction, all contributing to high blood pressure. Additionally, studies have shown that intermittent hypoxia facilitates the increased expression of hypoxia-induced factors (HIFs) and pro-inflammatory cytokines, further exacerbating inflammation and hypertension [60].

SDB and HTN are associated with chronic inflammation, a critical factor in the relationship between these conditions. Studies have found that irregular hypoxia triggers various inflammatory processes, releasing interleukin-6 (IL-6), tumor necrosis factor-α (TNF-α), and C-reactive protein (CRP) cytokines. These cytokines contribute to endothelial dysfunction, increased arterial stiffness, and further inflammation—all significant factors in developing HTN. Additionally, inflammation promotes monocytes and macrophages to accumulate and become functionally active within the vascular wall, which is crucial for atherogenesis [61,62,63]. Understanding this mechanism underscores the importance of addressing inflammation in patients with OSA who also have HTN.

During apneic episodes in OSA, attempts to inhale against an occluded airway produce large negative intrathoracic pressure, augment right ventricular preload, and shift the interventricular septum, impairing left ventricular filling. The increased transmural pressure also raises afterload, thereby reducing stroke volume and transiently lowering cardiac output. These abrupt hemodynamic perturbations, coupled with sympathetic overdrive, predispose to hypertension, arrhythmias, and heart failure [64,65]. Gradually, these hemodynamic stresses lead to the development of sustained hypertension. Moreover, mechanical stress caused by changes in intrathoracic pressure may cause organic damage, such as left ventricular hypertrophy, to the cardiovascular system, making the management of HTN in OSA patients even more challenging.

Frequent arousals from sleep, coupled with intermittent hypoxemia, lead to the activation of the sympathetic nervous system (SNS) in OSA. In addition, the fluctuations in intrathoracic pressure during obstructive events lead to alterations in arterial wall stretch, which in turn modify baroreceptor sensitivity and contribute to a dysregulated autonomic response [66]. This cascade increases sympathetic activity compounds, such as norepinephrine, which causes vasopressor effects, tachycardia, and elevated blood pressure. Increased sympathetic activity in OSA patients contributes to the onset of new HTN and other cardiovascular conditions, such as cardiac arrhythmias and myocardial ischemia. Additionally, sympathetic activation leads to renal sodium retention and fluid overload, further contributing to hypertension. This connection between sympathetic overactivity and HTN highlights the importance of addressing the SNS when managing OSA and HTN.

**Figure 3:**
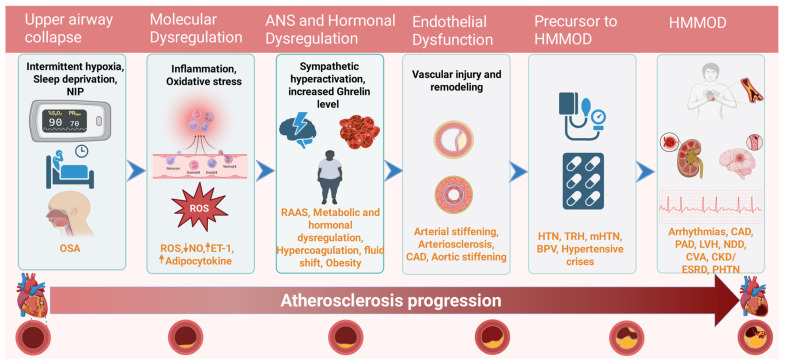
Stepwise progressive pathogenesis of hypertension leads to multi-organ dysfunction in OSA, even with an increasing number of medications. Early initiation of positive airway pressure therapy is crucial in preventing the progression of OSA-driven organ dysfunction. Abbreviations: HMMOD: hypertension mediated multi-organ damage, ANS = autonomic nervous system, RAAS = renin angiotensin aldosterone system, ROS = reactive oxygen species, NO = nitric oxide, OSA = obstructive sleep apnea, HTN = hypertension, TRH = treatment resistant hypertension, mHTN = masked hypertension, BPV = blood pressure variability, CAD = coronary artery disease, PAD = peripheral artery disease, LVH = left ventricular hypertrophy, NDD = neurodegenerative disorder, CVA = cerebrovascular accident, CKD = chronic kidney disease, ESRD = end stage renal disease, PHTN = pulmonary hypertension, NIP = negative intrathoracic pressure, ET-1 = endothelin 1; ↑ = increase; ↓ = decrease.

Endothelial dysfunction, a well-established pathophysiological mechanism of HTN, is closely linked to the vascular effects of IH and ROS, which are features of OSA. Hypoxia can result in damage to the endothelium, which is a crucial part of blood vessels that regulates vascular tone. This damage leads to a decrease in the availability of nitric oxide, a vasodilator, and an increase in the synthesis of endothelin-1 (ET-1), a vasoconstrictor [67]. This dysregulation is due to the predominance of vasoconstrictor forces over vasodilator forces, resulting in reduced endothelium-dependent vasodilation, increased vascular resistance, and, consequently, the development of HTN. Furthermore, endothelial dysfunction plays a significant role in promoting the progression of atherosclerosis, thrombosis, and vascular remodeling, which leads to HTN and HMMOD in OSA patients despite using multiple medications (Figure 3).

### 5.3. Clinical Features of OSA-Related Hypertension

In patients with OSA, hypertension is often characterized by specific characteristics that distinguish it from essential hypertension. OSA-related hypertension is typically severe, continuous, and resistant to known antihypertensive therapies. Most patients diagnosed with OSA exhibit higher blood pressure levels throughout the day, with significant rises during sleep due to repeated apneic episodes. The intermittent hypoxia and arousal-induced sympathetic activation associated with OSA lead to a persistent increase in sympathetic tone, contributing to a sustained elevation of blood pressure. OSA also leads to several hemodynamic and physiological alterations that result in sustained BP variability (BPV), an independent risk factor for cardiovascular disease and target organ damage beyond mean BP [68].

The distinctive feature of hypertension associated with OSA is the abnormal fluctuation in blood pressure throughout the day, especially at night. In healthy individuals, blood pressure follows a biologically determined 24 h cycle, with the lowest levels occurring during sleep. However, OSA patients often exhibit reduced or absent nocturnal dipping, a phenomenon known as non-dipping. Non-dipping is typically linked to increased cardiovascular disease and mortality, as it indicates the cardiovascular system’s inability to adequately lower blood pressure during rest, resulting in sustained arterial pressure and increased left ventricular workload [69]. Additionally, patients with OSA face the issue of nocturnal hypertension, where blood pressure tends to rise during sleep, causing cardiovascular strain. This nocturnal hypertension is directly related to apneic events, where the resulting hypoxia and arousals trigger surges in blood pressure that persist throughout the night and extend into the daytime.

OSA is a significant risk factor for TRH, and many TRH patients have inadequately managed OSA [70]. In the Heart Biomarker Evaluation in Apnea Treatment (HeartBEAT) study, which included 284 participants, those who were on an intensive blood pressure-control regimen (defined as three or more antihypertensive medications, including a diuretic) showed a higher prevalence of resistant elevated blood pressure if they had severe OSA compared to moderate OSA (58.3% vs. 28.6%, *p* = 0.01) [71]. The odds ratio (OR) of having resistant elevated blood pressure was four times higher for participants with severe OSA (OR 4.1, 95% CI: 1.7–10.2) [71]. This study’s findings suggest that untreated OSA may lead to poor blood pressure control despite an intensive antihypertensive regimen.

**Figure 4:**
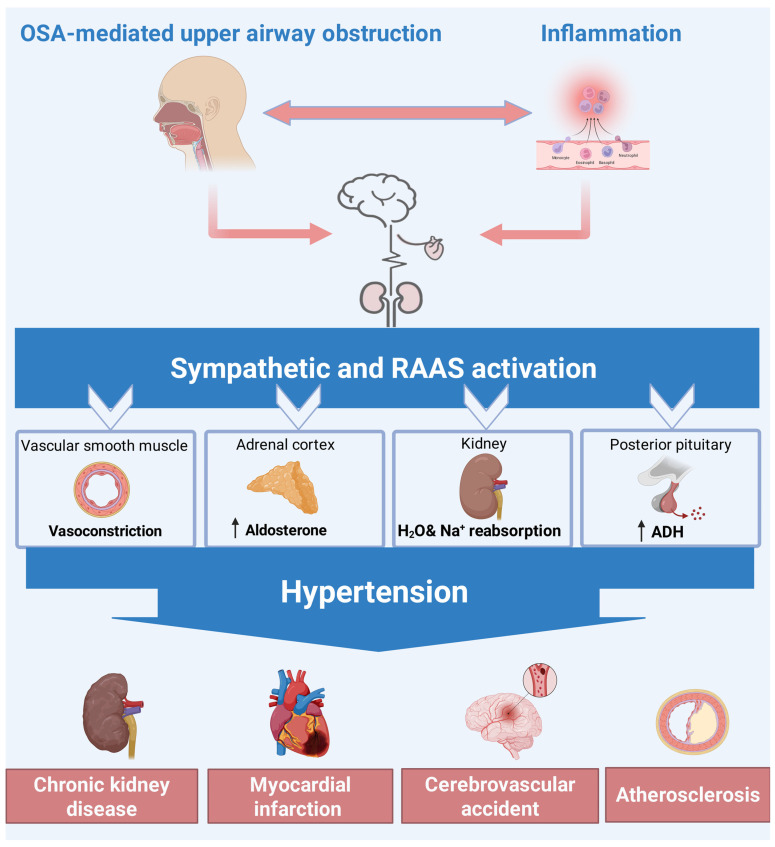
Pathogenesis of OSA-mediated treatment-resistant hypertension and multi-organ damage. OSA-driven hypoxia triggers a downstream cascade that leads to hypertension-mediated multiorgan dysfunction. Abbreviations: ADH = antidiuretic hormone; H_2_O = water; Na^+^ = sodium; ↑ = increase.

The pathophysiology linking OSA to TRH includes persistent sympathetic overactivity, aldosterone overproduction, increased arterial stiffness, and endothelial dysfunction. Aldosterone overproduction leads to impaired salt excretion, increased water–sodium retention, and nocturnal fluid shift towards the neck, which can cause pharyngeal edema and enhance airway collapse during sleep [71]. The coexistence of OSA with TRH complicates management because standard antihypertensive medications may not effectively control blood pressure. There are several antihypertensives for lowering blood pressure, but early CPAP therapy is the most effective treatment for OSA with hypertension (Table 4). CPAP is also the most effective therapy to lower blood pressure in patients with OSA-related TRH, highlighting the need for OSA screening and management in this patient population. In patients with TRH, untreated OSA increases the risk of cardiovascular events such as stroke, kidney disease, pulmonary hypertension, portal hypertension, heart attack, and heart failure (Figure 4). Beyond these hemodynamic sequelae, untreated OSA—and the resistant hypertension it often perpetuates—creates a markedly arrhythmogenic milieu, simultaneously destabilizing cardiac electrophysiology. OSA also substantially impacts cardiac rhythm: it promotes AF by remodeling the atria and increases the difficulty of AF management; it also predisposes to sudden cardiac arrest by creating an environment for lethal arrhythmias during sleep [72]. The mechanisms involve intermittent hypoxia, sleep fragmentation with surges of sympathetic activity, and intrathoracic pressure swings leading to structural heart changes [73]. Clinically, these insights reinforce the importance of diagnosing OSA in patients with atrial fibrillation, brady–tachy syndromes, or idiopathic ventricular arrhythmias. Managing OSA aggressively (e.g., with CPAP) is not only crucial for symptomatic relief, but it also becomes a cardioprotective strategy, improving AF ablation success, reducing arrhythmia recurrences, and possibly lowering the risk of nocturnal sudden death [74]. This multidisciplinary approach is reflected in current guidelines and expert recommendations to ensure patients receive comprehensive care addressing both their sleep disorder and cardiac condition [75].

The pharmacologic management of HTN in patients with OSA involves the use of antihypertensive medications such as Angiotensin-Converting Enzyme inhibitors (ACEi), Angiotensin II Receptor Blockers (ARBs), calcium channel blockers, and beta-blockers. However, these drugs are relatively ineffective in OSA-associated HTN due to sustained sympathetic tone and nighttime hypertension in untreated OSA patients. Diuretics, especially thiazide diuretics, are essential for managing HTN due to their effect on blood volume and arterial pressure. However, their utility is limited in managing OSA-related HTN since they do not address the intermittent hypoxia and airway occlusion typical of OSA [97]. In patients with obstructive OSA and resistant hypertension, hyperaldosteronism is prevalent, suggesting that aldosterone-driven fluid retention contributes to OSA pathophysiology. Mineralocorticoid receptor antagonists (MRAs) such as spironolactone and eplerenone block aldosterone’s effects, promoting natriuresis and attenuating the rostral fluid shift that occurs during sleep, thereby reducing nocturnal fluid accumulation in the neck with resultant pharyngeal edema and upper airway narrowing. Consistent with this mechanism, clinical studies and randomized trials have shown that add-on MRA therapy significantly lowers the apnea–hypopnea index and even reduces neck circumference, while improving blood pressure control in OSA patients (particularly in those with aldosterone excess). These improvements in OSA severity and cardiovascular parameters support the concept that aldosterone-mediated fluid retention is an important modifiable contributor to OSA and indicate that MRAs can serve as an effective adjunct treatment to mitigate OSA in appropriate patient populations [98,99].

Zilebesiran and IONIS-AGT-LR are novel RNA-based therapeutics that target angiotensinogen (AGT) synthesis in the liver, representing a possible shift in the management of hypertension in OSA, a phenotype characterized by RAAS hyperactivation, nocturnal hypertension, and aldosterone excess. Zilebesiran, RNA interference (RNAi) therapeutic, and IONIS-AGT-LR, an antisense oligonucleotide (ASO), lower circulating angiotensin I, angiotensin II, and aldosterone, mitigating nocturnal blood pressure surges, fluid retention, and airway edema, all of which contribute to the pathophysiology of OSA-related hypertension. In the KARDIA-1 trial, zilebesiran produced sustained 24 h SBP reductions (~15 mmHg at 6 months) with pronounced nighttime BP lowering, while IONIS-AGT-LR achieved >50% suppression of plasma AGT with favorable BP trends, particularly in resistant hypertension cohorts [79,100]. These long-acting, hepatocyte-targeted therapies offer the potential for biannual dosing, ensuring continuous BP control, preservation of renal autoregulation, and improved adherence, a critical advantage for OSA patients who require life-long RAAS inhibition. While direct trials in OSA populations are lacking, these agents address key mechanistic drivers of OSA-associated hypertension and may complement CPAP therapy by preventing nocturnal BP spikes and improving long-term cardiovascular outcomes. Further studies are warranted to assess their impact on apnea severity, aldosterone-driven airway remodeling, and nocturnal BP patterns in OSA cohorts.

Sodium–glucose cotransporter-2 (SGLT2) inhibitors, originally developed for type 2 diabetes, have demonstrated beneficial effects on the metabolic and cardiovascular risk profile of patients with OSA. These agents improve glycemic control, induce modest weight loss, and reduce blood pressure, and they significantly lower rates of cardiovascular and renal events in high-risk patients with or without coexisting OSA [101,102]. Notably, exploratory analyses of large outcome trials have reported a substantially lower incidence of new-onset OSA among participants treated with SGLT2 inhibitors compared to placebo (approximately 50% relative risk reduction) [103].

Emerging evidence also suggests that SGLT2 inhibition may directly attenuate OSA severity. Small studies in patients with OSA and coexisting heart failure or type 2 diabetes have shown significant reductions in the AHI and improved nocturnal oxygen saturation with SGLT2 therapy [104]. The putative mechanisms include the drugs’ pleiotropic metabolic effects, particularly weight reduction with decreased visceral adiposity, and their diuretic action, which, together, mitigate central obesity and nocturnal rostral fluid shifts that exacerbate upper-airway collapse [105]. Considering these findings, SGLT2 inhibitors are being considered as a promising adjunct in the management of OSA, especially in patients with obesity, type 2 diabetes, or heart failure, though dedicated trials are needed to confirm direct benefits on OSA severity and clinical outcomes [106].

CPAP is the current gold-standard therapy with a modest decrease in nocturnal and diurnal blood pressure by preventing upper airway collapse during sleep, thus reducing apnea and intermittent hypoxia. Studies have shown that CPAP is effective at lowering blood pressure. However, the magnitude of the decrease in blood pressure is relatively modest. Therefore, targeted antihypertensive medications are still required to achieve optimal blood pressure control. CPAP utilization was also associated with lower all-cause mortality and major adverse cardiovascular events (MACE) incidence, especially in older adults and patients with TRH [96,107]. Studies have shown that effective CPAP treatment in patients with moderate to severe OSA substantially reduces both day and night arterial blood pressure [108,109]. In a randomized parallel trial, when the difference between therapeutic and subtherapeutic nasal CPAP (nCPAP) was −3.3 mm Hg (95% CI −5.3 to −1.3; *p* = 0.0013), therapeutic nCPAP reduced mean arterial ambulatory blood pressure by 2.5 mm Hg, providing a significant vascular benefit, while subtherapeutic nCPAP increased blood pressure by 0.8 mm Hg [109]. If CPAP fails, surgical interventions such as hypoglossal nerve stimulation and expansion sphincter pharyngoplasty may be considered for selected patients with OSA. Uvulopalatopharyngoplasty (UPPP), a more invasive procedure, involves excising tissue in the throat to enlarge the airway and reduce apneic occurrences. However, these surgical approaches carry distinct risk factors and are appropriate only for carefully selected patients based on anatomical and physiological considerations [110]. Weight reduction remains a cornerstone of therapy for OSA-related hypertension, with bariatric surgery reserved for severely obese patients who require more aggressive metabolic intervention. In parallel, lateral sleeping positions can help reduce airway collapse by shifting gravitational effects on the pharynx and oral appliances, such as mandibular advancement devices, further support airway patency, underscoring a multifaceted approach that integrates lifestyle modification, positional therapy, and targeted interventions for optimal blood pressure control and improved sleep parameters [111,112].

## 6. Conclusions

Despite the availability of effective lifestyle modifications and pharmacologic treatments that can reduce blood pressure, hypertension control rates remain unsatisfactory worldwide. This systematic review underscores the complex interplay between hypertension and OSA, which contributes to the development and progression of multi-organ dysfunction and heightens the risk of adverse cardiovascular outcomes. These findings emphasize the need for early detection strategies and a multimodal treatment approach that integrates risk stratification with tailored management plans. Screening for OSA in patients with hypertension is warranted due to its high prevalence and its role in promoting blood pressure variability and resistance to antihypertensive therapy. Optimal care should involve both pharmacologic agents and nonpharmacologic measures, such as CPAP therapy and weight reduction. Although conventional antihypertensive therapies remain the cornerstone of management, emerging data suggest that additional targeted strategies may benefit select patients with OSA-related hypertension, particularly in the context of suboptimal CPAP adherence. However, much of the current evidence is derived from observational studies or randomized trials of limited size and duration. Accordingly, there is a pressing need for large-scale, prospective, and methodologically rigorous randomized studies to confirm these findings and refine treatment algorithms.

## Figures and Tables

**Table 1:** ABPM cutoff values for hypertension and non-dipping profile.

ABPM Parameter	Hypertension Threshold	Non-Dipping Criterion
24 h Mean BP	≥130/80 mmHg	
Daytime (awake) Mean BP	≥135/85 mmHg	
Nighttime (sleep) Mean BP	≥120/70 mmHg	
Nocturnal BP Drop		Normal dip: 10–20% fall Non-dipper: <10% fall Extreme dipper: >20% fall Reverse dipper: nighttime BP > daytime

**Table 2:** Key polysomnography parameters in OSA and severity criteria.

PSG Parameter	Definition and Measurement	OSA Diagnostic Threshold/Severity
**Apnea-Hypopnea Index (AHI)**	Number of apneas + hypopneas per hour of sleep (events/hour). An apnea is a ≥10 s breathing cessation; a hypopnea is a ≥30–50% airflow reduction with desaturation ≥3% or arousal	Primary metric for OSA diagnosis. AHI ≥ 5/h indicates OSA (with symptoms). Severity is classified as: Mild OSA if AHI 5–14; moderate OSA if AHI 15–29; severe OSA if AHI ≥ 30.
**Oxygen Desaturation Index (ODI)**	Number of oxygen desaturations ≥3% (or ≥4%) per hour of sleep (events/hour), reflecting frequency of oxygen drops. ODI is often reported with a 4% criterion in sleep studies.	Correlates with AHI and OSA severity. ODI is a surrogate for hypoxic burden. For example, ODI ≥ 5/h is abnormal and roughly corresponds to AHI ≥ 5. An ODI ≥ 15/h suggests at least moderate OSA, and ODI ≥ 30/h correlates with severe OSA. (ODI values show ~84–94% accuracy in predicting corresponding AHI severity cut-offs.) Higher ODI indicates more frequent or deeper desaturations, which are linked to cardiovascular risk.
**Arousal Index**	Number of EEG arousals per hour of sleep. An arousal is an abrupt shift in EEG to wake or lighter stage (≥3 s), often following an apnea/hypopnea.	Reflects sleep fragmentation. OSA causes frequent arousals from sleep due to respiratory events. A normal adult arousal index is on the order of <10–15/h (though it increases with age). OSA patients often have markedly elevated arousal indices proportional to AHI (e.g., an AHI of 30/h typically produces dozens of arousals per hour). A high arousal index contributes to non-restorative sleep and daytime sleepiness.
**Sleep Architecture**	Distribution of sleep stages (N1–N3 non-REM stages and REM sleep) and sleep continuity measures (total sleep time, sleep efficiency). PSG reports the percentage of time spent in each stage and sleep efficiency (% of time in bed asleep)	OSA-related alterations: OSA disrupts normal architecture. Normal adult sleep: ~5% stage N1, ~50% N2, ~20% N3 (slow-wave sleep), ~20–25% REM, with sleep efficiency >85%. In OSA, there is often an increase in light sleep (N1, N2) and a reduction in deep N3 and REM sleep due to recurrent arousals. Severe OSA can significantly reduce slow-wave and REM sleep percentages. Sleep efficiency may be reduced (<80%) because frequent awakenings and arousals fragment the sleep. These disruptions in architecture improve with effective OSA treatment (REM and N3 rebound with CPAP therapy)

**Table 3:** Studies used for data extraction.

Author	Year	Study Design	Sample Size	Population Characteristics	SDB and Hypertension Measures	Key Findings
Amin et al. [30].	2008	Comparative observational study	140	Children with SDB; otherwise, healthy	Apnea-hypopnea index (AHI), 24-h ambulatory blood pressure	SDB is independently associated with increased morning BP surge, BP load, and 24-h ambulatory BP. It is also associated with left ventricular remodeling, highlighting increased cardiovascular morbidity.
O’Connor et al. [31].	2009	Prospective cohort study	2470	Adults aged 40+ without baseline hypertension	AHI, incident hypertension over 5 years	The relationship between AHI and incident hypertension was attenuated after adjusting for BMI. The association was not statistically significant, but a modest association cannot be excluded.
Tanigawa et al. [32].	2004	Cross-sectional study	1424	Japanese men aged 40–69 in rural and urban communities	3% Oxygen Desaturation Index (ODI), SBP/DBP	3% ODI level is positively associated with higher SBP and DBP. The association was more evident among overweight individuals, suggesting a role of SDB in HTN development among Japanese men.
Sánchez-de-la-Torre et al. [33].	2015	Experimental study	100 (subsample)	Adults with resistant hypertension and obstructive sleep apnea (OSA)	BP response to CPAP treatment, miRNA profiles	A pre-CPAP treatment cluster of 3 plasma miRNAs predicted BP responses to CPAP in patients with Resistant hypertension and OSA.
Witkowski et al. [34].	2011	Interventional study	10	Patients with resistant hypertension and sleep apnea	BP measurements, AHI, polysomnography	Renal sympathetic denervation significantly lowered BP and improved sleep apnea severity.
Horne et al. [35].	2011	Comparative observational study	141	Children with varying severities of SDB and nonsmoking controls	BP during sleep and wake, categorized by sleep states	SDB, regardless of severity, was associated with elevated BP during both sleep and wake states compared to controls.
Peppard et al. [36].	2000	Prospective, Population-Based	709	Participants from the Wisconsin Sleep Cohort Study, general population sample	AHI measured via polysomnography; hypertension defined as BP ≥ 140/90 mm Hg or use of medications	A dose-response association between SDB and hypertension was observed, independent of confounding factors.
Reid et al. [37].	2011	Cross-Sectional	60	Pregnant women with gestational hypertension (n = 34) and healthy pregnant women (n = 26)	Frequency of SDB assessed via polysomnography; respiratory disturbance index ≥ 5	Women with gestational hypertension had a higher frequency of SDB compared to healthy women; the impact of SDB and obesity on this condition is unclear.
Li et al. [38].	2020	Prospective, Population-Based	11,623	U.S. Hispanic/Latino participants from the Hispanic Community Health Study/Study of Latinos	AHI ≥ 5 for SDB; insomnia assessed with Women’s Health Initiative Insomnia Rating Scale ≥ 9; incident hypertension and diabetes	SDB was associated with higher odds of incident hypertension and diabetes. Insomnia was associated with incident hypertension, with a stronger association among men.
Minic et al. [39].	2014	Retrospective, Cross-Sectional	52	Patients with WHO group 1 pulmonary arterial hypertension (PAH)	Prevalence of SDB assessed via PSG; Epworth Sleepiness Scale (ESS) for subjective sleepiness	High prevalence of SDB in PAH patients. Older age and higher ESS scores were predictive of SDB.
Facco et al. [40].	2017	Prospective Cohort	3705	Nulliparous women, assessed early and mid-pregnancy	AHI ≥ 5 used to define SDB; hypertensive disorders of pregnancy (HDP), including preeclampsia and gestational diabetes mellitus (GDM) measured	SDB in pregnancy is independently associated with an increased risk of preeclampsia, hypertensive disorders, and GDM. The relationship between AHI and these outcomes is exposure–response dependent.
Ulrich et al. [41].	2013	Randomized, Double-Blind, Cross-Over	23	Patients with pre-capillary pulmonary hypertension (PH) and SDB	Nocturnal oxygen therapy (NOT) and acetazolamide effects on 6-min walk distance (MWD), SDB, and hemodynamics	NOT improved 6 MWD and SDB along with hemodynamic improvements in patients with pre-capillary PH. No significant improvement in quality of life or exercise performance was observed with acetazolamide.
Spiesshoefer, et al. [42].	2021	Prospective, Cross-Sectional	71	Patients with pre-capillary pulmonary hypertension (PH) and 35 matched controls	Prevalence and severity of OSA and nocturnal hypoxemia; Assessed via overnight cardiorespiratory polygraphy, lung function, HCVR, and cardiac MRI	High prevalence of OSA in PH patients, with significant associations between SDB severity and reduced 6 MWD, NT-proBNP levels, and right ventricular function. The clinical impact of these findings warrants further investigation.
Eskandari et al. [43].	2018	Randomized Controlled Trial (Three-way Crossover)	13	Male patients with hypertension and moderate to severe OSA; mean age: 64 ± 7 years; BMI: 29 ± 4 kg/m^2^; AHI: 37 ± 23 events/h	Blood pressure (office and 24-h), arterial stiffness, polygraphic sleep study data, AHI, venous bicarbonate concentration	AZT alone and AZT + CPAP reduced blood pressure and AHI. CPAP alone did not significantly affect blood pressure. AZT is a potential drug therapy for OSA with hypertension.
Zhang et al. [44].	2024	Observational Study with PCA and Metabolomic Analysis	3299 (Discovery) + 1522 (Validation)	Hispanic Community Health Study/Study of Latinos; OSA characterized by age, gender, respiratory event frequency, and sleep quality	SDB-related measures, metabolite risk scores (MRS), incident hypertension and diabetes	SDB is associated with distinct metabolomic profiles. SDB-related metabolite signatures are linked with 6-year incident hypertension and diabetes, suggesting potential biomarkers for SDB risk stratification.
Kato et al. [45].	2019	Observational Study	52	Elderly adults with SDB; mean age: 69.6 ± 4.0 years; no impairment in daily living activities	Apnea/hypopnea index (AHI), minimum oxygen saturation (SpO_2_), hypertension (measured via questionnaire and BP value)	Nocturnal hypoxia (SpO_2_ < 90%) and hypertension negatively impacted cognitive function. HTN was the most significant factor for cognitive decline in tasks like WCST and N-back task.

Abbreviations: AZT = acetazolamide, AHI = apnea-hypopnea index, ODI = oxygen desaturation index, SDB = sleep disordered breathing, BMI = basic metabolic index, BP = blood pressure, SBP = systolic blood pressure, DBP = diastolic blood pressure, miRNA = micro–Ribonucleic Acids, CPAP = continuous positive airway pressure, PAH = pulmonary arterial hypertension, SpO_2_ = oxygen saturation, MRI = magnetic resonance imaging, HCVR = Hypercapnic Ventilatory Response, PH = pulmonary hypertension, 6 MWD = 6-Minute Walk Distance, OSA = obstructive sleep apnea, NT-proBNP = N-terminal pro B-type natriuretic peptides, PCA = Principal Component Analysis. WCST = Wisconsin Card Sorting Test.

**Table 4:** Treatment for hypertension in OSA patients. An effective treatment regimen should include options that address key mechanisms known to be driven by OSA.

	Treatment	Mechanism	Benefits	Limitations	Selected RCT and Meta-Analysis (MA) in Patients with OSA and HTN
**Pharmacologic Therapies**	Antihypertensives ACE inhibitors, ARBs, calcium channel blockers, beta-blockers	Reduction in vasoconstriction and sympathetic overactivity	Reduces BP, prevents cardiovascular events.	Limited efficacy in OSA due to persistent airway obstruction; potential negative effects on sleep quality due to frequent awakening from nocturnal diuresis.	RCT 2000: Atenolol and Hydrochlorothiazide were more effective than amlodipine, losartan, and enalapril in lowering blood pressure in OSA patients with HTN [76]. VALSAS trial: Valsartan induced a fourfold decrease in mean 24-h BP in OSA patients by inhibiting angiotensin II and preventing vasoconstriction [77].
	Diuretics and Sympatholytic	Lowers arterial pressure and volume particularly in OSA-mediated fluid retention and sympathetic overdrive	Reduces preload and lowers BP, particularly effective in patients with fluid retention.	Ineffective as standalone treatment; does not address airway obstruction or intermittent hypoxia.	RCT 2024: in OSA patients with HTN, Guanfacine lowered 24-h blood pressure more than HCTZ, but HCTZ still showed a significant effect [78].
	RNA Interference (RNAi) Precision medicine	Targets gene expression to reduce angiotensin II production, lowering BP.	Promising for OSA-related HTN and TRH; potential shift in HTN management.	Novel therapy with limited clinical experience; requires more long-term data.	RCT: in OSA patients with TRH, 3 identified miRNAs predict BP response to CPAP [33]. KARDIA-1 trial: RNA interference with single subcutaneous dose of zilebesiran significantly lowers BP up to 6 months [79].
	SGLT2-Inhibitors	Promotes natriuresis and glycosuria, reducing plasma volume and BP.	Cardioprotective, addresses metabolic and cardiovascular issues; benefits patients with comorbid diabetes or HF.	Not a primary treatment for OSA; efficacy may vary between patients.	EMPA-REG OUTCOME trial: empagliflozin leads to a significant reduction in BP for Patients with Presumed TRH not specific to OSA-related HTN [80]. CANVAS trial: canagliflozin leads to significant BP lowering, not specific to OSA-related HTN [81]. DAHOS trial: in HFrEF with OSA patients, dapagliflozin significantly reduced blood pressure and apnea-hypopnea index (AHI) [82].
**Non-CPAP Therapies**	Oral/Nasal Appliances	Devices like soft-palate lifts, tongue-retaining devices, and mandibular advancement appliances reposition anatomical structures to alleviate airway obstruction.	Reduces apneic episodes, improves BP control, alternative for CPAP intolerance.	Variable efficacy; not as effective as CPAP therapy.	MA: Provent is a disposable nasal device that reduces AHI and improves oxygen saturation in patients with OSA. The data on BP benefits are limited. It is currently discontinued [83].
	Expansion Sphincter Pharyngoplasty, Hypoglossal Nerve Stimulation	Surgical and minimally invasive procedures to stabilize and enlarge the airway or stimulate upper airway muscles.	Reduces OSA severity and improves BP in some patients intolerant to standard treatments.	Invasive, costly, with potential complications; reserved for specific cases.	CARDIOSA-12 trial: Hypoglossal Nerve Stimulation (HGNS) therapy did not improve BP and other CV measures [84]. STAR trial: HGNS reduces AHI and a number of oxygen desaturations [85]. 22 April 2025 2:23:00 PM
	Uvulopalatopharyngoplasty (UPPP), Renal Denervation	UPPP removes excess throat tissue to enlarge the airway; renal denervation impairs renal sympathetic nerves to control BP.	UPPP reduces apneic episodes; renal denervation shows potential in reducing BP in TRH patients.	Mixed BP results; UPPP may not address core OSA pathophysiology; renal denervation is invasive and a last resort.	SYMPLICITY HTN-3: Renal denervation reduced BP in TRH patients; in a post hoc analysis, OSA patients appeared to be responsive to renal denervation therapy [86,87]. RADIANCE trials: renal denervation showed a significant BP reduction in TRH; but this was not specific to OSA-related HTN [86,87,88,89].
**Lifestyle Interventions**	Weight Loss	Reduces OSA severity, lowers BP, and improves overall cardiovascular health through lifestyle changes, medications, or bariatric surgery.	Effective in reducing both OSA and HTN severity; bariatric surgery is an option for severe obesity.	Requires long-term commitment; bariatric surgery carries risks and is suitable for selected patients only.	SURMOUNT-OSA: in patients with moderate-to-severe OSA plus Obesity, Tirzepatide reduced AHI, BMI, and SBP [90]. GATEWAY: Bariatric surgery reduced BP in obese patients with HTN [91]. MA: A decline in body weight in patients on GLP-1 RA resulted in a decrease in SBP [92]. 22 April 2025 2:23:00 PM
**Gold Standard Therapy**	CPAP Therapy	Provides continuous airflow to prevent upper airway collapse during sleep, reducing apnea, hypoxia, and sympathetic activity.	Reduces both nocturnal and daytime BP; prevents cardiovascular events such as heart attack and stroke.	Tolerance issues in some patients; adherence is critical for effectiveness.	SAVE trial: Early CPAP adherence is associated with modest reductions in BP and significant cardiovascular benefits in OSA patients [93]. HIPARCO trial: Optimal adherence to CPAP restores BP dipping pattern and significantly reduces daytime and nighttime blood pressure in patients with OSA and TRH [94,95]. MA: CPAP treatment significantly reduced BP in OSA patients with TRH [96]. 22 April 2025 2:23:00 PM

Abbreviations: RCT = randomized controlled trials; MA = meta-analysis; CV = cardiovascular; SAVE = Sleep Apnea cardioVascular Endpoints; HIPARCO = Hypertension and Obstructive Sleep Apnea Reduction by CPAP; CPAP = continuous positive airway pressure; BP = blood pressure; SBP = systolic blood pressure; OSA = obstructive sleep apnea; TRH = treatment resistant hypertension; HTN = hypertension; ACE = Angiotensin-Converting Enzyme inhibitors; ARBs = Angiotensin II Receptor Blockers; GLP-1 RA = GLP-1 receptor agonists; VALSAS = Valsartan Treatments on Arterial Blood Pressure in obstructive sleep apnea syndrome; GATEWAY = Gastric Bypass to Treat Obese Patients With Steady Hypertension; SURMOUNT-OSA = Semaglutide Unabated Reduction in Metabolic Outcomes and Obesity via Novel Treatment-Obstructive sleep apnea; AHI = apnea hypopnea index, BMI = basic metabolic index; RADIANCE = Renal Denervation in Arterial Hypertension; SYMPLICITY HTN-3 = Sympathetic Renal Denervation in Patients with Treatment-Resistant Hypertension; STAR = Stimulation Therapy for Apnea Reduction; CARDIOSA-12 = Cardiovascular Endpoints for Obstructive Sleep Apnea With Twelfth Nerve Stimulation; DAHOS = Dapagliflozin in Heart Failure and Obstructive Sleep Apnea Syndrome; HFrEF = heart failure reduced ejection fraction; SGLT2 = Sodium-Glucose Cotransporter 2; CANVAS = CANagliflozin cardioVascular Assessment Study; EMPA-REG OUTCOME = Empagliflozin Cardiovascular Outcome Event Trial in Type 2 Diabetes Mellitus Patients.

## Data Availability

The datasets used and analyzed during the current study are available from the corresponding author on reasonable request.
